# A Review of the Impact of Maternal Obesity on the Cognitive Function and Mental Health of the Offspring

**DOI:** 10.3390/ijms18051093

**Published:** 2017-05-19

**Authors:** Laura Contu, Cheryl A. Hawkes

**Affiliations:** School of Life, Health and Chemical Sciences, Science, Technology, Engineering and Mathematics (STEM) Faculty, Open University, Walton Hall, Milton Keynes MK7 6AA, UK; laura.contu@open.ac.uk

**Keywords:** maternal obesity, high fat diet, offspring brain, mental health, cognitive function, epigenetics

## Abstract

Globally, more than 20% of women of reproductive age are currently estimated to be obese. Children born to obese mothers are at higher risk of developing obesity, coronary heart disease, diabetes, stroke, and asthma in adulthood. Increasing clinical and experimental evidence suggests that maternal obesity also affects the health and function of the offspring brain across the lifespan. This review summarizes the current findings from human and animal studies that detail the impact of maternal obesity on aspects of learning, memory, motivation, affective disorders, attention-deficit hyperactivity disorder, autism spectrum disorders, and neurodegeneration in the offspring. Epigenetic mechanisms that may contribute to this mother–child interaction are also discussed.

## 1. Introduction

Despite being classified as an epidemic by the World Health Organization in 2007, rates of obesity in many countries continue to rise unabated. Recent estimates of global obesity suggest that approximately 37% of men and 30% of women are overweight or obese [1]. In some countries, such as Kuwait, Libya, and Tonga, the estimated prevalence of obesity among women aged 20 years and older is close to or over 60% [1]. Rates of maternal obesity have risen in parallel [2,3,4], due to both greater numbers of obese pregnant women and excess weight gain during pregnancy [5,6]. 

Maternal obesity influences the health of both the mother and child. Pre-natal complications include increased risk of miscarriage, pre-eclampsia, gestational diabetes, and thromboembolism [7]. In addition, maternal obesity negatively impacts upon placental, embryonic, and fetal growth and increases the risk of post-partum complications [6,7,8]. Experimental and clinical evidence arising from research into the developmental origins of health and disease (DoHAD) suggests that maternal obesity also has long-lasting consequences on the health of the offspring. It is now established that maternal obesity is associated with increased body-mass index (BMI) of offspring across infancy [9], adolescence [10], and into adulthood [11,12]. Obesity during gestation is also linked with increased risk of coronary heart disease, diabetes, stroke, asthma, and premature death in adult offspring [13,14,15]. 

Increasingly, DoHAD studies are looking at the relationship between maternal obesity and the offspring brain. Although much of this work has focused on the neuronal mechanisms that contribute to offspring obesity, there is mounting—but sometimes contradictory—evidence for an influence of maternal obesity on the cognitive function and mental health of the offspring. As increasing numbers of children are born to overweight and obese mothers, there is a pressing need to clarify the relationship between the gestational environment and long-term mental health, including the cellular and molecular mechanisms that underlie individual susceptibility or resistance.

Among these mechanisms, epigenetic regulation of gene expression in the fetal brain is gaining increasing attention. Epigenetic mechanisms are those that influence gene expression independently of alterations to the underlying DNA sequence. Currently identified mechanisms include DNA methylation, chromatin remodelling, histone modification, and modulation via non-coding RNA. Although most epigenetic studies in human offspring of obese mothers have focused on changes in peripheral tissues, there is compelling evidence from animal studies to suggest that DNA methylation patterns are disrupted in the brains of adult offspring exposed to a high fat diet during the prenatal period [16,17]. The identification of altered DNA methylation in mental illnesses such as depression and schizophrenia [18,19], support a putative relationship between maternal obesity, epigenetic dysregulation, and mental health in later life.

This review will summarize findings from human and animal studies on the long-term impact of maternal obesity on cognitive function in the offspring brain, as well as the predisposition towards psychiatric and neurodegenerative diseases. It will also outline some of the epigenetic mechanisms identified to date that may underlie this mother–child interaction.

## 2. Cognitive Function and Mental Health in Offspring Born to Obese Mothers

### 2.1. Animal Studies

Most animal models induce maternal obesity by feeding female animals calorically-dense foods, including high fat or “cafeteria” diets. Feeding schedules are typically initiated before (e.g., pre-pregnancy obesity) or at the start of gestation and/or lactation to distinguish the effects of pre- and peri-natal high fat exposure on brain plasticity and development [20,21]. In most cases, offspring are maintained on a low fat control diet, although some experiments have also fed the offspring the same high fat diet as the mother to determine the effect of sustained high fat diet exposure [22,23,24,25,26]. Investigations into the impact of maternal obesity or high fat feeding on offspring cognition and mental health in animal models have focused predominantly on anxiety, depression, learning and memory, and motivation, with some newer studies also examining the susceptibility to developing neurodegenerative diseases. 

### 2.2. Depression and Anxiety

Studies examining the effect of maternal obesity on offspring anxiety have reported mixed results. Initial investigations using non-human primates reported that young adult female, but not male, macaques born to mothers fed a high fat diet showed increased anxiety to novel and threatening objects [27]. These findings were supported by rodent experiments that found more anxiolytic behaviors in the elevated plus maze and open field test in 1–3-month old male and/or female offspring born to dams fed a high fat diet before mating and during gestation and lactation compared to offspring born to dams fed a control diet [28,29,30,31]. However, weanling (e.g., post-natal day 23–45) rodents born to obese mothers or those fed a cafeteria diet during lactation exhibit lower levels of anxiety [32,33]. Decreased anxiety has also been reported in young adult rats whose mothers were fed a high fat diet before, but not during pregnancy [20], as well as in offspring of dams fed a cafeteria diet during lactation only [34]. Balsevich et al. reported that three-month old male offspring born to obese mothers showed lower levels of anxiety compared to those born to lean mothers, but higher anxiolytic symptoms at 12 months of age [35]. These results suggest that pre- vs. post-natal exposure to a high fat diet may result in different susceptibility to anxiety in the offspring, which may also vary according to sex and brain maturation. This may also be influenced by diet-induced variations in the degree of maternal licking and grooming [33], which is known to influence anxiety responses in offspring via epigenetic modifications in glucocorticoid receptor expression [36]. A recent report in piglets that found no association between prenatal high fat feeding and anxiety suggests that such effects may also be species-specific [37]. Although fewer studies have been carried out looking at the relationship between maternal obesity and depression, reports to date suggest that young adult male rodents exposed to a high fat diet either in utero [35,38] or during lactation [39] show higher rates of depression-like symptoms than those born to lean dams. 

### 2.3. Learning and Memory 

Assessments of the effect of maternal obesity on offspring learning and memory have largely focused on the acquisition and retention of spatial memory. Bilbo and Tsang [28] reported that young adult male and female rats born to dams fed a diet high in either saturated or trans fats performed significantly better on the Morris water maze task compared to control rats. Better spatial memory performance was also observed in three-month old offspring born to obese *Peromyscus* mouse dams [40], as well as in 11–15-week old piglets exposed to a high fat diet either during the prenatal or pre- and post-natal periods [41,42]. However, another study found that spatial memory performance in the Barnes maze was decreased in 4- and 10-week old male offspring of obese mothers compared to those born to lean mothers [43]. This effect was also observed in offspring exposed to a high fat diet throughout both the pre- and postnatal period, who showed decreased retention times, higher escape latencies, and less time spent in the target quadrant of the water maze, suggesting impaired spatial memory performance [22,24,25,26]. Similarly contradictory findings have been described using the novel object recognition task, with one experiment reporting decreased exploration of the novel object in young adult male—but not female—offspring of obese dams [44] and another reporting higher initial novel exploration by male offspring exposed to a cafeteria diet during lactation [21]. Again, these discrepancies may relate to the timing of dietary exposure, sex, and age of offspring at testing. 

### 2.4. Motivation and Attention

One hypothesis for the interrelationship between maternal obesity and increased risk of obesity in the offspring is that this early life exposure to over-nutrition leads to a blunted reward response and increased motivation for consumption of palatable food. This hypothesis is supported by data showing that male rats born to dams fed a high fat diet before or during pregnancy make more responses and have lower latency to reach an appetitive reward [20,45]. This behaviour is associated with altered dopaminergic and µ-opioid receptor expression and signaling [46,47,48]. However, this hypothesis has been challenged by recent studies showing that male and/or female offspring born to dams fed a high fat diet during gestation are less motivated for appetitive rewards than offspring born to dams fed a control diet [17,49]. Young adult mice exposed to a high fat diet in utero also demonstrate higher impulsivity and attentional deficits in the five-choice serial-reaction time and attentional set-shifting tasks compared to control animals [17,49,50].

### 2.5. Dementia and Neurodegeneration

Individuals who have mid-life hypertension, diabetes, and hyperlipidemia are at increased risk of developing Alzheimer’s disease (AD) [51]. Given the association between maternal obesity and metabolic disease in adult offspring, a few animal studies have also examined the impact of prenatal high fat feeding on AD-like cognitive impairment and pathology. Martin et al. reported impairments in memory acquisition and retention in the triple transgenic mouse model of AD (3xTgAD) in female mice exposed to a high fat diet during gestation and lactation at both 6- and 12-months of age [52]. This corresponded to a 50% increase in phosphorylated tau-positive neurons, one of the pathological hallmarks of AD, in the hippocampus of 12-month old mice exposed to the high fat diet versus control animals. Clearance of the β-amyloid (Aβ) peptide, another pathological marker of AD, is impaired in the brains of adult male mice born to obese mothers [23]. Aged offspring born to obese Tg2576 mice that express the Swedish mutation in the human amyloid precursor protein [53] also develop increased Aβ pathology compared to offspring of lean Tg2576 mice [54]. These preliminary studies suggest that the impact of maternal obesity on the cognitive function of the offspring extends not only across the lifespan, but may also predispose towards increased risk of developing neurodegenerative diseases like AD, vascular dementia, and multiple sclerosis [15,55,56].

## 3. Human Studies

The impact of maternal obesity on cognitive function and the development of psychiatric disorders in human offspring has recently been reviewed [57,58,59]. In general, analyses of longitudinal, prospective, and observational studies support an association between maternal BMI and poorer cognitive performance, as well as increased risk of developing depression and anxiety. The relationship between maternal obesity and offspring risk of developing attention-deficit hyperactivity disorder (ADHD) and autism spectrum disorder is currently less clear. 

### 3.1. Depression, Anxiety, and Internalizing and Externalizing Behaviours

Children who have a positive disposition are more likely to report higher mid-life satisfaction and are at lower risk of developing mental illness in adulthood [60,61]. Recent studies have demonstrated that four-month old infants born to mothers who reported consuming more than the recommended allowance of saturated fat have lower surgency scores than those born to mothers with low total fat intake [62]. Similarly, maternal consumption of a Mediterranean diet during pregnancy was negatively associated with child externalising problems [63], supporting an effect of maternal diet on behavioural outcomes in their offspring. Maternal BMI is a predictor of poorer psychosocial development, as evidenced by higher externalizing symptoms and lower social competence in children aged 5–47 months [64]. Data from the large cohort studies suggest that maternal pre-pregnancy BMI is also associated with increased risk of internalizing problems, including symptoms of withdrawal, depression, and anxiety in 3–8-year-old children [65,66,67]. Moreover, children born to obese mothers had less rapid decreases in internalizing scores with time, such that internalizing problems identified at 8-years old increased through to age 17 [68]. 

### 3.2. Cognitive Function and Intelligence Quotient (IQ)

More than a dozen observational studies have been conducted looking at the association between maternal BMI and cognitive function of the offspring during infancy and adolescence. These studies largely support a negative correlation between pre-pregnancy maternal obesity and child IQ [69,70,71,72,73,74,75,76,77,78], including poorer motor, spatial, and verbal skills [69,76,79,80]. In some cases, lower cognitive performance is also observed in children as a function of maternal weight gain during pregnancy [76,77,81], although the opposite has also been reported [82]. Indeed, lower IQ and higher risk of intellectual disability are also related to low maternal BMI [76,78,83], suggesting that there is a U-shaped association between maternal weight and offspring cognitive performance. Although these findings should be interpreted cautiously due to potentially unidentified confounds and ongoing maturation of the brain into early adulthood, evidence supporting a relationship between low childhood IQ and increased risk of developing psychiatric disorders in adulthood [84] supports a role for maternal obesity on offspring mental health across the life-course. 

### 3.3. Attention-Deficit Hyperactivity Disorder (ADHD)

Elevated maternal BMI has also been positively associated with difficulties with emotional regulation and high inattention scores in five-year-old children as reported by teacher, but not parental ratings [67]. A large cohort study of over 12,000 children also reported higher teacher-ranked scores of ADHD symptoms in 7–8-year-olds born to mothers with both high pre-pregnancy BMI and excess gestational weight gain [85]. However, a study by Tonda and Salisberry [86] reported increased ADHD in children born to obese Caucasian, but not African-American women, suggesting that racial background may also play a role in offspring susceptibility. In addition, a population-based survey across more than half a million 9–17-year-olds found that although high pre-pregnancy BMI increased the risk of ADHD in the offspring, the association was lost in full sibling comparisons [87], indicating that unidentified familial confounds may also contribute to ADHD risk.

### 3.4. Autism Spectrum Disorders

Mixed findings have also been reported for the association between maternal weight and risk of autism spectrum disorders. Large cohort studies from Canada, U.S.A., and the U.K. have found that children born to obese mothers were more likely to be diagnosed with autism [88,89,90]. This risk is further exacerbated in offspring of obese women who gain excess weight during pregnancy [77]. However, matched sibling analysis of over 6000 children did not report a significant association between maternal obesity and offspring risk of autism [91]. Similarly to IQ, children born to mothers who are both underweight and obese appear to be at greater risk of developing autism spectrum disorders [92]. 

## 4. Epigenetic Mechanisms Induced by Maternal Obesity in the Offspring Brain

Multiple mechanisms have been suggested to underlie the effects of maternal high fat feeding on the offspring brain. Alterations in levels of brain-derived neurotrophic factor and Notch signaling genes, decreased proliferation of neuronal progenitor cells and abnormal synaptic stability, reduced and dendrite length and branching have all been reported in animal offspring born to obese mothers [43,93,94,95]. Although gene expression is particularly sensitive to epigenetic regulation during brain development and maturation, surprisingly little work has been done to investigate the putative epigenetic mechanisms by which maternal obesity influences offspring cognition and mental health. Among the classical epigenetic mechanisms, DNA methylation is an attractive target for investigation because levels of folic acid, a co-factor in the production of the principle methyl donor methionine [96], are decreased in the amniotic fluid of obese pregnant women [97]. Indeed, Vucetic et al. [16] found that DNA methylation was decreased in the promoter regions of the μ-opioid receptor genes as well as globally in brain regions associated with reward such as the ventral tegmental area (VTA), prefrontal cortex (PFC), and nucleus accumbens (NAc) of young male offspring born to obese mothers. These changes persist up to a year after birth [48]. Supplementation of maternal high fat diet with methyl donors during gestation and lactation attenuated the diet-induced increase in dopamine transporter mRNA levels in the VTA and NAc of male offspring and restored levels of DNA methylation in the PFC and NAc [48]. Recently, the same group has demonstrated that prenatal methyl supplementation can also reverse deficits in motivation and attentive behaviour of young adult offspring exposed to a high fat diet during early life [17,49]. Separate to DNA methylation, Edlow et al. [98] have reported the differential expression of 37 microRNAs, including those related to inflammation and vascular health, in the brains of embryonic mice born to mothers fed a high fat vs. control diet. 

To date, no work has been done out to determine epigenetic changes in the brains of human offspring born to obese mothers. However, alterations in the degree of DNA methylation in cord blood [99] and microRNA levels in amniotic fluid [100] have been reported in human studies of maternal obesity, supporting a putative role for these epigenetic mechanisms in mediating the effects of maternal obesity on the offspring brain.

## 5. Conclusions

The gestational environment plays a critical role on the long-term health of the fetus. Results from animal studies examining the impact of maternal obesity on the cognitive performance and behaviour of the offspring are currently mixed. Inter-experimental differences in diet composition (e.g., purified high fat or cafeteria diet), initiation (e.g., before or at the start of breeding), and duration (e.g., throughout gestation and/or lactation) of dietary manipulation make direct comparisons of results difficult. It is likely that timing of dietary intervention, age, sex, species, and maternal-pup interactions all contribute to the resistance or susceptibility of the offspring to developing anxiety, depression, memory impairments, and changes in motivation and attention. Current data from human cohort studies support a negative association between high maternal BMI and child IQ, as well as risk of developing depression and anxiety. A clear association between maternal obesity and increased risk of ADHD and autism spectrum disorder in the offspring has not yet been established and may be influenced by additional biological or social factors. To date, most of the work on the associated epigenetic mechanisms have focused on DNA methylation, and differential patterns of methylation have been identified in the brains of offspring born to lean vs. obese mothers in rodent models. Undoubtedly, new and exciting findings will soon help to unravel the complex processes that influence the developmental programming of the brain.
